# Intelligent diagnosis of ossicular chain malformations on CT: development and clinical efficacy of a cascaded AI framework

**DOI:** 10.3389/fbioe.2026.1731385

**Published:** 2026-07-06

**Authors:** Yanjun Gao, Rui Wang, Junle Yang, Yishan Li, Liangliang Shang, Xinyi Meng, Qing Zhou, Feng Shi, Xiaoping Wu, Jian Yang

**Affiliations:** 1 Department of Diagnostic Radiology, The First Affiliated Hospital of Xi’an Jiaotong University, Xi’an, Shaanxi, China; 2 Xi’an Key Laboratory of Metabolic Disease Imaging, Xi’an No.3 Hospital, Affiliated Hospital of Northwest University, Xi’an, Shaanxi, China; 3 Department of Research and Development, Shanghai United Imaging Intelligence Co., Ltd., Shanghai, China; 4 Department of Radiology, Xi’an Central Hospital, Xi’an, Shaanxi, China; 5 Shaanxi Engineering Research Center of Computational Imaging and Medical Intelligence, Xi’an, Shaanxi, China; 6 Xi’an Key Laboratory of Medical Computational Imaging, Xi’an, Shaanxi, China

**Keywords:** artificial intelligence, ear ossicles, machine learning, ossicular chain malformations, temporal bone

## Abstract

**Objective:**

The aim of this study was to develop and validate a cascaded artificial intelligence (AI) framework using a segmentation–discrimination strategy for automated detection of ossicular chain malformations (OCM) on computed tomography scans.

**Methods:**

Patients diagnosed with OCM between January 2009 and January 2023, along with healthy controls, were retrospectively enrolled. A coarse-to-fine nnU-Net framework was applied for automated segmentation of the auditory ossicles. Separate discrimination models were developed for the malleus, incus, and stapes, and subsequently integrated to generate a patient-level model. The performance of the proposed framework was compared with deep learning networks, a volume-threshold algorithm, and by radiologists. The temporal external validation was conducted on data from multiple sites within Shaanxi Province, using the same CT vendors. The discriminatory capacity of each ossicular-level model was assessed using area under the curve (AUC), sensitivity, specificity, and accuracy.

**Results:**

A total of 2,462 temporal bone computed tomography scans (training set, n = 1,302; test set, n = 556; validation set, n = 604) were analyzed. The cascaded network demonstrated AUC values of 0.962, 0.930, and 0.931 for the malleus, incus, and stapes, respectively. At the patient level, accuracy, sensitivity, and specificity were 0.874, 0.949, and 0.800, respectively, surpassing the performance of ResNet, DenseNet, the volume-threshold algorithm, and junior radiologists, while equaling that of senior radiologists. In temporal validation, the network maintained robust performance, with AUC values of 0.972, 0.973, and 0.963, and patient-level metrics of 0.960 (accuracy), 0.974 (sensitivity), and 0.947 (specificity).

**Conclusion:**

The cascaded AI framework using a segmentation–discrimination strategy demonstrated high performance in identifying patients with diverse types of OCMs on computed tomography scans. This approach has the potential to support radiologists in the diagnostic evaluation of OCMs.

## 1 Introduction

**FIGURE 1:**
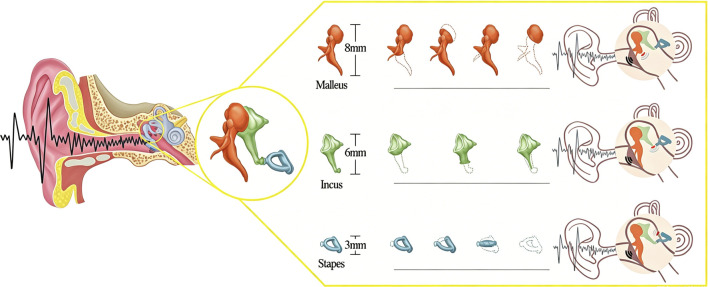
Schematic illustration of sound wave transmission through the ossicular chain. This figure illustrates how sound waves travel from the external auditory canal through the ossicular chain to the inner ear, highlighting how ossicle deformities disrupt sound transmission. The malleus (orange), incus (green), and stapes (blue) are color-coded for clarity in depicting normal and abnormal structures.

The auditory ossicles, comprising of the malleus, incus, and stapes, represent the smallest bones in the human body (Martonos et al., 2023). These bones are interconnected to form the ossicular chain, which transmits sound waves to the inner ear. Ossicular chain malformations (OCM) represent the most common type of middle ear malformation and can cause significant hearing impairment, potentially resulting in deafness. Common manifestations of OCM include diverse developmental anomalies of the malleus, incus, and stapes, as depicted in Figure 1. Clinical detection remains challenging due to the small size of the ossicles and the heterogeneity of malformations. For individuals with conductive hearing loss related to OCM, surgical reconstruction is often required. Such procedures depend on accurate diagnostic evaluation and individualized surgical planning (Tang et al., 2018; Yamashita et al., 2018). Accurate diagnosis of OCMs is therefore critical for effective clinical decision-making.

Temporal bone computed tomography (CT) scans are considered the gold standard for assessing OCM (Jager et al., 2005). Preoperative high-resolution CT, combined with ossicular chain reconstruction techniques demonstrated an 88.9% concordance rate with intraoperative diagnoses, although this approach is relatively time-consuming (Gao et al., 2012). Increasing demand for imaging studies, combined with a limited number of radiologists specializing in otolaryngology, may increase the risk of diagnostic errors (Bruno et al., 2015).

Advances in computer-aided diagnosis, particularly the use of artificial intelligence (AI) in medical imaging, have demonstrated considerable potential for disease detection and classification (Yu et al., 2023; Liu et al., 2021). Although AI-assisted technologies have demonstrated maturity in the evaluation of large organs, their application to microstructures such as the auditory ossicles remains limited. Emerging evidence indicates the diagnostic potential of deep learning in otology. Prior studies have validated the efficacy of deep learning in diagnosing otosclerosis using temporal bone CT and have demonstrated potential for detecting cochlear malformations (Fujima et al., 2021; Emin et al., 2024; Li et al., 2024). Notably, previous research confirmed the feasibility of deep learning-based ossicular segmentation, providing a foundational reference for AI-assisted diagnosis of OCM (Wang et al., 2022).

A cascaded AI model was developed for the diagnosis of OCM, using a multi-tiered architecture in which the output of each preceding model served as the input for subsequent models to facilitate both segmentation and discrimination of the ossicles. This stepwise strategy improved detection accuracy for small auditory structures by progressively refining candidate regions and reducing computational complexity by decomposing the diagnostic task into manageable subtasks. The objective of this study was to refine and validate a segmentation–discrimination approach for the automated identification of OCM on temporal bone CT scans, thereby establishing a framework for AI-assisted classification of microstructural abnormalities.

## 2 Materials and methods

### 2.1 Patients

Approval for this retrospective study was obtained from the institutional review boards of the participating institutions, and the requirement for informed consent was waived.

Patients diagnosed with OCM from January 2009 to January 2023 at the Third Hospital of Xi’an and Xi’an Central Hospital were included. The reference standard was established through multidisciplinary consensus by a panel consisting of two experienced radiologists (with >10 years of experience in head and neck imaging) and two senior otologists (with >10 years of clinical experience). The final diagnosis for each case was determined based on the comprehensive integration of clinical symptoms (e.g., conductive hearing loss), audiometry results (pure-tone audiometry, tympanometry), and CT imaging findings, supplemented by intraoperative findings when available. The final diagnosis for each case was determined when at least three of the four panel members reached agreement. In cases where consensus could not be reached, the case was discussed in a joint meeting until a unanimous decision was achieved. This approach reflects real-world clinical practice, where diagnosis is typically reached through synthesis of multiple data sources rather than reliance on a single modality. We acknowledge that this introduces an incorporation bias, meaning the AI model may learn to replicate the interpretive patterns of the radiologists involved in establishing the reference standard. Given that the goal of this study is to develop an assistive tool that can achieve diagnostic performance comparable to that of expert radiologists, this bias is acceptable as long as the model demonstrates strong consistency with the established clinical consensus. The exclusion criteria were: (1) patients with middle ear tumors, significant trauma, or vascular complications as confirmed by a senior radiologist; (2) patients who had undergone ear surgery prior to temporal bone CT examination; and (3) patients with severe image artifacts or suboptimal image quality.

Additionally, healthy controls were selected at a 1:1 ratio. The inclusion criteria were: (1) individuals who underwent temporal bone CT examination; and (2) individuals with normal ossicular chain structure and function. The exclusion criteria were identical to those applied for the patient group.

An external temporal validation set was constructed using data collected at Xi’an Central Hospital, The First Affiliated Hospital of Xi’an Jiao Tong University and, Xi’an First Hospital, between February 2024 and February 2026 with CT scanners from the same vendors as the primary cohort, applying the same criteria, for the purpose of evaluating the performance of the model.

### 2.2 Image data collection and preprocessing

Temporal bone CT examinations were performed using CT scanners (Brilliance iCT, Philips; SOMATOM Force, Siemens) with the following parameters: tube voltage, 120 kV; tube current, 200 mAs; field of view, 250 mm × 250 mm; reconstruction matrix, 1024 × 1024; reconstruction interval, 0.2–0.3 mm; rotation speed, 0.4 s/360°; and collimation, 0.625 mm. All scans were acquired with a scanning length of 1.5 cm, extending from the lower edge of the external auditory canals to the upper edge of the petrous bones.

To enhance model performance and training efficiency, a series of preprocessing steps were applied to the original temporal bone CT data. (1) Resampling: Volumes had variable slice thicknesses (0.3–1.0 mm). All volumes were resampled to an isotropic voxel spacing of 0.5 × 0.5 × 0.5 mm^3^ using trilinear interpolation to ensure spatial consistency. (2) Cropping: Images were cropped based on the region of interest to exclude irrelevant background information. (3) Intensity normalization: Hounsfield unit (HU) values were first clipped to [−2000, 4,000] and linearly normalized to [−1, 1]. Subsequently, Z-score normalization was applied using the mean and standard deviation computed from the training set to center and scale the data. The order was corrected to avoid contradiction.

Original DICOM temporal bone CT data from the training and test sets were imported into ITK-SNAP 3.2 software. Images were manually annotated layer by layer under the bone window by two radiologists with expertise in otolaryngology. One radiologist performed the segmentation, while the second radiologist reviewed the annotations to ensure completeness and accuracy.

### 2.3 Auditory ossicles automatic segmentation

U-Net, an encoder–decoder architecture introduced in 2015, substantially advanced the field of medical image segmentation (Ronneberger et al., 2015). In this study, the open-source segmentation framework No-new U-net (nnU-Net) (Isensee et al., 2021; Kharaji et al., 2024), was used, recognized for its superior performance in biomedical image segmentation tasks. This self-adaptive U-Net framework demonstrates considerable flexibility, dynamically adjusting network structure and parameter settings according to specific segmentation tasks.

**FIGURE 2:**
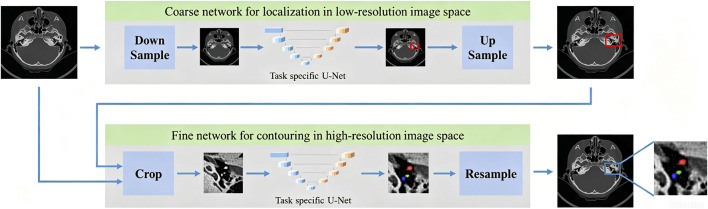
Architecture of the coarse-to-fine cascaded segmentation framework for auditory ossicles. The coarse network first localizes the auditory ossicle region in low-resolution image space by down-sampling the input CT image and generating a rough region of interest. The localized region is then transferred to the fine network, where the image is cropped and segmented in high-resolution image space for precise contouring of the ossicles. Finally, the segmentation results are resampled to the original image space.

In this study, a coarse-to-fine cascaded nnU-Net framework was used for automated segmentation of the auditory ossicles. As depicted in Figure 2, two task-specific U-Net models were applied in a cascaded manner. Specifically, in the low-resolution image space, the general positions of the malleus, incus, and stapes were initially located. Subsequently, the edges of the three auditory ossicles were finely delineated in a relatively high-resolution image space. The automated segmentation framework for the auditory ossicles was implemented in PyTorch 1.7.0 and executed using a single Nvidia Tesla V100 graphics processing unit.

### 2.4 Radiomics model at malleus/incus/stapes-level

#### 2.4.1 Feature extraction

Radiomic features were categorized into seven groups. A total of 105 features were extracted from the original normalized images, with most parameters set to their default values. In accordance with ISBI guidelines, feature extraction was performed using the open-source software PyRadiomics 2.2.0 (https://pyradiomics.readthedocs.io/en/latest/). Detailed data is provided in Electronic Supplementary Material 1.

#### 2.4.2 Feature selection

To reduce the risk of overfitting and improve computational efficiency, a stepwise feature selection strategy was applied: (1) Mann-Whitney U test (p < 0.05); (2) Spearman correlation (if |r| > 0.9, the feature with higher mean absolute correlation was removed), retaining 62 features; (3) MRMR (Minimum Redundancy Maximum Relevance) selecting the top 30 features; (4) LASSO regression with 10-fold cross-validation to tune λ (grid search from $10^{-4}$ to 10 ). The optimal λ = 0.025 (lambda.min) yielded 8 non-zero-coefficient features for the final model.

#### 2.4.3 Establishment of machine learning-based models

**FIGURE 3:**
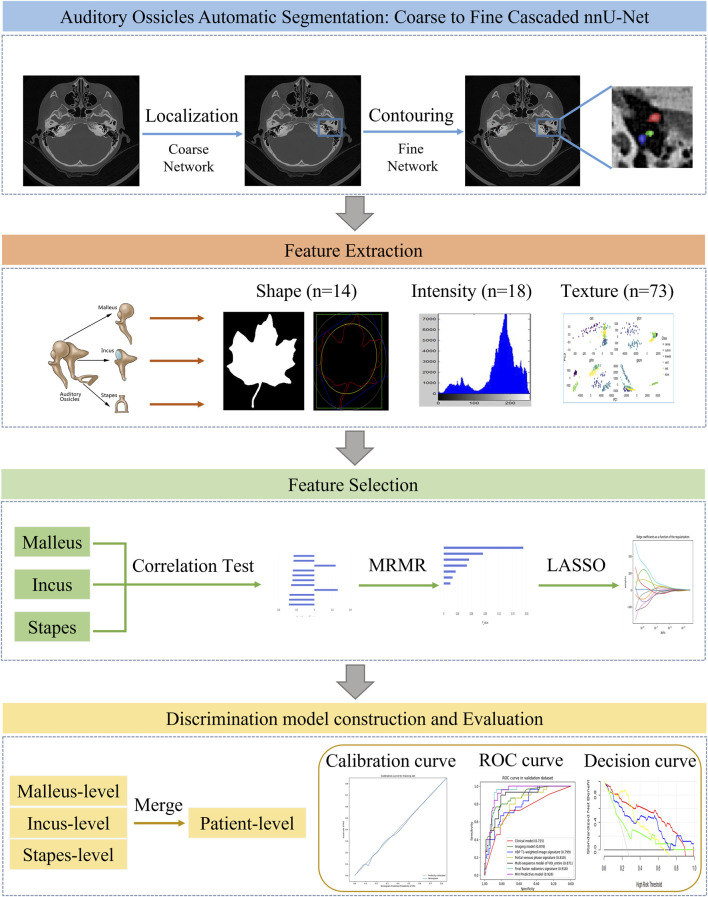
The overall flowchart of our study. A coarse-to-fine cascaded nnU-Net was used to automatically segment the auditory ossicles on CT images. Radiomic and morphologic features were then extracted and selected from the malleus, incus, and stapes. Finally, discrimination models were built at the ossicle and patient levels, and their performance was evaluated.

Machine learning classifiers, specifically random forest (RF), logistic regression (LR), and decision tree (DT), were used to develop identification models for abnormal auditory ossicles at the malleus, incus, and stapes levels, respectively (Breiman, 2001; Vapnik, 1999; Alves et al., 2021). Additionally, the grid search method was used to automatically identify the optimal hyperparameter values for these machine learning classifiers. As depicted in Figure 3, the patient-level model for abnormal auditory ossicles was generated by integrating the discrimination results from the malleus, incus, and stapes level models.

### 2.5 Visual evaluation by a radiologist

Conventional diagnostic evaluations of all CT images in the test set were independently performed by four radiologists. Two radiologists were junior physicians without specialized training in temporal bone CT interpretation, while the other two were senior radiologists. All evaluators were blinded to both the final clinical diagnoses and the output of the cascaded AI framework.

### 2.6 Performance evaluation and statistical analysis

The performance of the automatic auditory ossicles segmentation model was evaluated using the Dice similarity coefficient (DSC), 95% Hausdorff distance (HD95), and average surface distance (ASD) between the automatically segmented and manually delineated masks.

The discrimination ability of the models at the malleus, incus, and stapes levels was assessed using several metrics, including area under the curve (AUC), sensitivity, specificity, and accuracy. Additionally, receiver operating characteristic (ROC) curves and calibration curves were generated to evaluate the agreement between model predictions and observed outcomes.

The calibration curves included Brier score calculations for each model, with lower scores indicating improved prediction accuracy. Additionally, decision curve analysis was conducted to assess the clinical utility of the models by quantifying their net benefit across varying probability thresholds. All metrics and curves were calculated separately for the training and test datasets. Finally, overall performance at the patient level was evaluated by calculating sensitivity, specificity, and accuracy, which were compared among the different models. Deep learning frameworks were used for the discrimination of abnormal auditory ossicles. Specifically, multi-task frameworks, including Res-Net and Dense-Net, were used to simultaneously identify abnormalities in the malleus, incus, and stapes (He et al., 2016; Huang et al., 2017). A volume-threshold approach was applied to categorize the malleus, incus, and stapes, with the optimal threshold determined via grid search to maximize accuracy in the training cohort.

For external validation, assessing model generalizability to independent datasets, temporal validation was conducted using data from the same sources but from distinct time periods relative to the development cohort. Identical evaluation metrics were applied to ensure comparability. Paired McNemar’s tests were performed to compare diagnostic disagreement between the model and radiologists at different levels for binary outcomes. To account for multiple comparisons, a Bonferroni correction was applied by multiplying the original p-values by the number of comparisons (k = 12, corresponding to 4 radiologists × 3 ossicles). A corrected p-value < 0.05 was considered statistically significant.

Statistical analyses were performed using IBM SPSS Statistics 20.0 and Python 3.7.0. ROC curves, calibration curves, Brier loss, and decision curves were generated using the open-source library Scikit-learn 0.23.2 in Python. Figure 3 depicts the overall flowchart of the study.

## 3 Results

A total of 59 participants were excluded due to the presence of other middle ear diseases or suboptimal image quality. Ultimately, 929 participants were included, comprising of 462 patients with OCM and 467 patients with normal bilateral ossicular chains. In total, 1,858 eligible temporal bone CT scans were analyzed, with each participant undergoing bilateral temporal bone CT. The scans were randomly assigned to a training group (n = 1,302) and a test group (n = 556) in a 7:3 ratio, ensuring no participant overlap between groups. Additionally, the external validation set included 302 participants, comprising of 152 patients with OCM and 150 healthy controls with normal ossicular chains, resulting in a total of 604 temporal bone CT scans for analysis. Detailed data is provided in Table 1.

**TABLE 1:** The detailed number of the training, testing and validation dataset.

Characteristic	Training	Testing	Validation	Total
Demographics				
Age (years), mean ± SD	19.2 ± 12.34	20.6 ± 11.34	20.2 ± 12.50	19.8 ± 12.17
Sex, male (%)	342 (52.5%)	146 (52.5%)	159 (52.6%)	647 (52.6%)
Patient-level diagnosis				
Normal	327	140	150	617
Abnormal	324	138	152	614
Malformation types by ossicle (abnormal patients only)				
Malleus abnormality	126 (38.9%)	38 (27.5%)	35 (23.0%)	199 (32.4%)
Incus abnormality	172 (53.1%)	72 (52.2%)	78 (51.3%)	322 (52.4%)
Stapes abnormality	188 (58.0%)	77 (55.8%)	92 (60.5%)	357 (58.1%)
Combined (≥2 ossicles)	162 (50.0%)	49 (35.5%)	53 (34.9%)	264 (43.0%)
Ossicle-level distribution (left + right ears combined)				
Malleus-level normal	1,156	492	557	2,205
Malleus-level abnormal	146	64	47	257
Incus-level normal	993	425	466	1884
Incus-level abnormal	309	131	138	578
Stapes-level normal	976	414	451	1841
Stapes-level abnormal	326	142	153	621

Percentages for malformation types are based on abnormal patient counts; patients may have multiple ossicle involvements. Ossicle-level counts represent the total number of ossicles (left and right ears combined).

The segmentation performance of the cascaded model on the test set is presented in Table 2. Specifically, DSC values were > 0.9 for the malleus and incus and 0.8–0.9 for the stapes; 95% HD95 ranged from 1–2 mm for all ossicles, except the left incus, which measured 3 mm; ASD was <1 mm for all ossicles, except the right stapes, which measured 1.14 mm.

**TABLE 2:** The segmentation performance of our model in the testing dataset.

Side	Auditory ossicles	DSC	HD95	ASD
Left	Malleus	0.933	1	0.22
	Incus	0.937	3	0.55
	Stapes	0.851	1.2	0.22
Right	Malleus	0.939	1	0.31
	Incus	0.942	1	0.32
	Stapes	0.842	2.0	1.14

DSC, dice similarity coefficient; HD95, Hausdorff distance; ASD, average surface distance.

**FIGURE 4:**
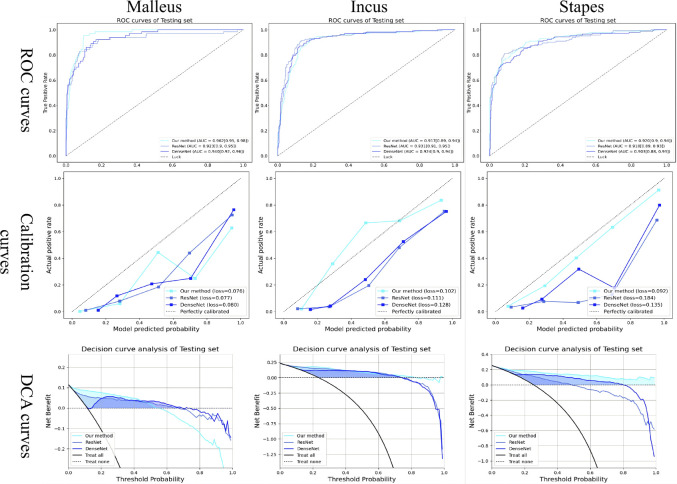
The various curves of our method comparing to other algorithms. The first row: the ROC curve comparison of the models in the testing dataset; The second row: the calibration curve comparison of the models in the testing dataset; The third row: the DCA curve comparison of the models in the testing dataset.

An analysis was conducted to evaluate the discriminatory performance of various machine learning models in identifying abnormal auditory ossicles, summarized in Electronic Supplementary Material 2. The LR model was selected for distinguishing abnormal malleus, and the RF model was chosen for identifying abnormal incus and stapes, representing the optimal models for auditory ossicle discrimination. The cascaded model outperformed ResNet, DenseNet, and volume-threshold methods, achieving significantly higher AUCs for malleus (0.962; 95% CI: 0.95–0.98) and stapes (0.920; 95% CI: 0.90–0.94) detection (all *p* < 0.05), although no significant inter-model differences were observed for the incus (*p* > 0.05). At the patient level, the model demonstrated balanced performance, with a sensitivity of 0.949, specificity of 0.800, and accuracy of 0.874. Comparative analyses (Table 3; Figure 4) confirmed superior discriminatory power relative to conventional deep learning approaches.

**TABLE 3:** The performance comparison of our method with other algorithms both in the training dataset and the testing dataset.

Level	Model	Dataset	Sensitivity	Specificity	Accuracy	AUC
Malleus	Our method	Training cohort	0.932	0.921	0.922	0.978
		Testing cohort	0.906 (0.896–0.942)	0.902 (0.897–0.924)	0.903 (0.872–0.941)	0.962 (0.944–0.977)
	Res-net	Training cohort	1.000	0.983	0.985	1.000
		Testing cohort	0.688	0.949	0.919	0.923
	Dense-net	Training cohort	1.000	0.977	0.979	1.000
		Testing cohort	0.828	0.921	0.910	0.940
	Volume_10.43	Training cohort	0.507	0.981	0.928	--
		Testing cohort	0.484	0.982	0.924	--
Incus	Our method	Training cohort	0.997	0.925	0.942	0.994
		Testing cohort	0.855 (0.813–0.875)	0.852 (0.792–0.861)	0.853 (0.832–0.879)	0.917 (0.889–0.932)
	Res-net	Training cohort	0.990	0.905	0.925	0.996
		Testing cohort	0.885	0.854	0.862	0.931
	Dense-net	Training cohort	1.000	0.948	0.960	1.000
		Testing cohort	0.916	0.852	0.867	0.924
	Volume_14.48	Training cohort	0.401	0.956	0.824	--
		Testing cohort	0.427	0.951	0.827	--
Stapes	Our method	Training cohort	0.975	0.894	0.915	0.989
		Testing cohort	0.845 (0.805–0.870)	0.951 (0.913–0.978)	0.924 (0.901–0.965)	0.920 (0.880–0.941)
	Res-net	Training cohort	0.979	0.947	0.955	0.997
		Testing cohort	0.789	0.848	0.833	0.910
	Dense-net	Training cohort	1.000	0.777	0.833	1.000
		Testing cohort	0.894	0.679	0.734	0.903
	Volume_1.44	Training cohort	0.687	0.970	0.899	--
		Testing cohort	0.627	0.966	0.809	--
Patient	Our method	Training cohort	0.991	0.838	0.914	--
		Testing cohort	0.949	0.800	0.874	--
	Res-net	Training cohort	0.988	0.881	0.934	--
		Testing cohort	0.935	0.764	0.849	--
	Dense-net	Training cohort	1.000	0.673	0.836	--
		Testing cohort	0.971	0.564	0.766	--
	Volume	Training cohort	0.787	0.917	0.852	--
		Testing cohort	0.761	0.893	0.827	--

AUC, area under the curve. The data in parentheses are the 95% confidence intervals. The volume threshold of malleus, incus and stapes is shown in the column of ‘Model’.

Table 4 indicates that the cascaded model significantly outperformed junior radiologists in diagnostic accuracy for all ossicular assessments (malleus, incus, stapes; all *p* < 0.05), while matching the performance of senior radiologists for incus and stapes evaluations (*p* > 0.05), as confirmed by McNemar’s test.

**TABLE 4:** The performance comparison of radiologists at different experience levels on the test set.

Level	Model	Sensitivity	Specificity	Accuracy	P value^*^
Malleus	Radiologist 1	0.703 (45/64)	0.813 (400/492)	0.800 (445/556)	p = 0.008
	Radiologist 2	0.844 (54/64)	0.860 (423/492)	0.858 (477/556)	p = 0.036
	Radiologist 3	0.891 (57/64)	0.977 (445/492)	0.979 (502/556)	p = 0.038
	Radiologist 4	0.859 (55/64)	0.981 (436/492)	0.928 (491/556)	p = 0.048
Incus	Radiologist 1	0.702 (92/131)	0.831 (353/425)	0.800 (445/556)	p = 0.010
	Radiologist 2	0.779 (102/131)	0.819 (348/425)	0.809 (450/556)	p = 0.013
	Radiologist 3	0.840 (110/131)	0.913 (388/425)	0.896 (498/556)	p = 0.056
	Radiologist 4	0.847 (111/131)	0.871 (370/425)	0.865 (481/556)	p = 0.320
Stape	Radiologist 1	0.634 (90/142)	0.785 (325/414)	0.746 (415/556)	p = 0.000
	Radiologist 2	0.697 (99/142)	0.807 (334/414)	0.779 (433/556)	p = 0.001
	Radiologist 3	0.845 (120/142)	0.969 (401/414)	0.937 (521/556)	p = 0.700
	Radiologist 4	0.803 (114/142)	0.942 (390/414)	0.906 (504/556)	p = 0.120

Radiologists 1 and 2 were junior practitioners, while Radiologists 3 and 4 held senior positions.

^*^ Comparison between the cascaded model and each radiologist using McNemar’s test.

As presented in Table 5, the cascaded model demonstrated robust performance on the external validation set. It achieved high specificity and accuracy while maintaining excellent sensitivity across all ossicular levels. Specifically, sensitivity ranged from 0.862 to 0.925, specificity from 0.905 to 0.941, and accuracy from 0.907 to 0.941, confirming consistent diagnostic performance across the malleus, incus, and stapes.

**TABLE 5:** Performance of the cascaded model on the external validation set.

Level	Sensitivity	Specificity	Accuracy	AUC
Malleus	0.862	0.923	0.941	0.964
Incus	0.925	0.941	0.940	0.954
Stapes	0.898	0.905	0.907	0.933
Patient	0.967	0.926	0.958	--

AUC, area under the curve.

## 4 Discussion

The proposed method demonstrated high performance in identifying diverse types of OCM on CT scans, with accurate automated segmentation, making it suitable for clinical adjunctive evaluation. Furthermore, this work addresses a gap in AI-based diagnosis of OCM and achieves fully automated identification of abnormal auditory ossicles.

From the segmentation model performance results, the model demonstrated high accuracy and robustness in segmenting the malleus and incus on both the left and right sides. This performance is likely attributable to the relatively simple and stable anatomical structures of these ossicles, which facilitate feature learning and recognition. Segmentation performance indicated a slight decrease for the stapes on both sides, possibly due to its complex morphology. The performance of the segmentation model in this study was comparable to that of the 3D-V-Net model in accurately segmenting normal auditory ossicles, while demonstrating substantially improved accuracy in segmenting deformed ossicles (Wang et al., 2022). This enhancement may be attributed to the inclusion of a large number of malformed ossicle samples in the dataset, which exposed the model to a broader range of morphological variations during training and consequently improved its ability to identify abnormal ossicles.

Prior studies have consistently highlighted considerable challenges in stapes segmentation. Powell et al. used an atlas-based segmentation approach, reporting DSC values > 0.80 for both the malleus and incus, but notably lower values for the stapes (left: 0.58; right: 0.48) (Powell et al., 2017). Similarly, Ding et al. reported poor stapes segmentation performance (DSC = 0.36), despite achieving high accuracy for the malleus (0.83) and incus (0.84) (Ding et al., 2022). These findings collectively indicate that conventional segmentation techniques face considerable challenges in stapes delineation. Neves et al. used a ResNet-based approach, achieving an overall DSC of 0.87 for the entire ossicular chain (Neves et al., 2021). Performance improved substantially when segmenting each ossicle individually, as demonstrated in previous work using 3D-V-Net (Wang et al., 2022). This study achieved superior accuracy, even for malformed ossicles, by implementing a cascaded segmentation framework. These results indicate that the approach provides enhanced precision in ossicular chain segmentation, particularly for anatomically complex or pathological structures.

The method uses a highly accurate segmentation model of the auditory ossicles and utilizes grayscale statistics, shape, and texture features to construct the abnormality detection model. This strategy closely aligns with clinical approaches for identifying abnormalities, enhancing the effectiveness of the model in recognizing small structures. In addition, our model detects ossicular abnormalities based on bone density and morphological features extracted from the segmented ossicle region only, which excludes surrounding soft tissue. Although performance is comparable to deep learning techniques for the malleus and incus, the model demonstrates superior accuracy in identifying the complex morphology of the stapes, thereby providing improved diagnostic performance for ossicular abnormalities.

Deep learning models, through continuous convolution, expand their receptive field, which may expose them to irrelevant background features and affect the learning and classification of small structures. In addition, the volume-threshold model does not account for age, a critical factor in ossicle development, resulting in substantially lower performance. Numerous studies have explored the role of AI in otology, including the application of deep learning for delineating normal temporal bone structures, detecting a single type of ossicular chain sclerosis in CT scans, and using integrated deep learning approaches for diagnosing various conditions using otoscope images (Fujima et al., 2021; Li et al., 2020; Cha et al., 2019).

Regarding AI-assisted diagnosis of ossicle-related disorders, Fujima et al. proposed a deep learning algorithm for detecting ossicular chain sclerosis, along with GoogLeNet and ResNet, each achieving a diagnostic accuracy of 0.86 (Fujima et al., 2021). Despite these promising results, limitations such as small patient sample sizes—the aforementioned otosclerosis study included only 198 temporal bone CT images—must be acknowledged. Given the limited availability of comparable research, direct comparisons with other automated methods for detecting OCMs were not performed in this study.

It is noteworthy that the external validation set yielded higher accuracy and specificity than the internal test set. First, the internal test set contained older CT scans (up to 10 years old) with lower image quality, whereas the external validation set consisted of more recent scans. The model may benefit from cleaner inputs, a form of positive domain shift. Second, this may be partly attributable to random statistical variability given the finite sample size of the external set. Thus, the apparent superiority of external performance should not be overinterpreted; a larger, prospective external validation is needed to obtain more stable estimates.

The model can be integrated into PACS as a silent second reader, providing per-ossicle abnormality predictions within ∼3 s per case. It could save an estimated 40%–50% time reduction minutes per reading, especially for junior radiologists. Primary use cases include assisting less experienced readers and triaging emergency scans. Prospective time-motion and user acceptance studies are needed to validate these benefits.

This study has several limitations. Firstly, the reference standard in this study was established through multidisciplinary consensus, which incorporated CT imaging as one component of the diagnostic process. Most patients did not undergo surgery after CT examination; consequently, only 37 cases (8.0%) in the internal cohort had surgical confirmation. For the remaining cases without surgical confirmation, the ground truth is therefore partially derived from the same imaging modality that the AI model learns from. This introduces an incorporation bias, meaning the AI model may learn to replicate the interpretive patterns of the radiologists involved in establishing the reference standard. Given that the objective of this study is to develop an assistive tool that achieves diagnostic performance comparable to that of expert radiologists, this bias is acceptable as long as the model demonstrates strong consistency with the established clinical consensus. Future prospective studies with independent surgical validation would be valuable to further assess the model’s ability to detect malformations independently of expert interpretation. Secondly, the model has only been validated on a small amount of data from three hospitals, and has not yet undergone large-scale validation; its generalizability still needs to be strengthened. Factors that could contribute to performance variability include differences in tube voltage, slice thickness, reconstruction algorithms, patient positioning, and the prevalence of specific malformation subtypes. Thirdly, while our model is robust to moderate soft tissue opacification, extremely dense or extensive soft tissue that completely obscures ossicular boundaries could degrade segmentation. Future work should evaluate the model on such challenging cases. Fourthly, we did not apply any explicit imbalance handling techniques. Although the absolute number of abnormal ossicles in the training set was sufficiently large for the model to learn discriminative features, and our model performed well without any explicit imbalance correction, the imbalance may still affect generalizability to extremely rare subtypes. Finally, variations in imaging parameters across different CT devices and the subjectivity of manual annotations may introduce biases. Future work will focus first on optimizing the existing cascaded model to enhance the accuracy of automated segmentation and detection of OCMs. Subsequently, the functionality of the model will be expanded to enable identification and classification of OCM in complex cases, including those involving tumors or inflammation.

Concurrently, multicenter validation studies will be conducted to increase dataset diversity and improve computational efficiency. The model’s clinical utility will also be systematically evaluated, and its potential applications in the analysis of small-organ imaging will be further explored. We will provide a prediction webpage (URL: https://gateway.xa3yuan.com:5001) with expected launch date June 2026; access via username/password provided by the authors. Requests should be sent to [wangrui27ray@163.com]. The webpage and weights will be shared for academic research purposes only.

In summary, the segmentation-discrimination approach uses the nnU-Net framework for automated segmentation of auditory ossicles and incorporates machine learning models to detect abnormalities in the malleus, incus, and stapes. This method enables accurate and automated identification of abnormal auditory ossicles, providing substantial support for clinical decision-making. The technology has the potential to serve as a valuable diagnostic aid, particularly in medical institutions and radiology departments with limited expertise in temporal bone imaging.

## Data Availability

The original contributions presented in the study are included in the article/Supplementary Material, further inquiries can be directed to the corresponding authors.
