# Supplementary material for: Intelligent diagnosis of ossicular chain malformations on CT: development and clinical efficacy of a cascaded AI framework
Source: Front Bioeng Biotechnol. 2026 Jul 6;14:1731385. doi: 10.3389/fbioe.2026.1731385 (PMC13381513; doi:10.3389/fbioe.2026.1731385)
Supplement: Supplementary file 2 [file Table2.docx]

**Electronic Supplementary Material 2**

**The performance comparison of different machine learning models both in the training dataset and the testing dataset.**

| **Auditory ossicles** | **Model** | **Dataset** | **Sensitivity** | **Specificity** | **Accuracy** | **AUC（95%CI）** |
| --- | --- | --- | --- | --- | --- | --- |
| Malleus | RF | Training cohort | 0.986 | 0.908 | 0.917 | 0.992（0.99，1.00） |
|  |  | Testing cohort | 0.906 | 0.894 | 0.896 | 0.963（0.95，0.98） |
|  | **LR** | Training cohort | 0.932 | 0.921 | 0.922 | 0.978（0.97，0.98） |
|  |  | Testing cohort | 0.906 | 0.902 | **0.903** | 0.962（0.95，0.98） |
|  | DT | Training cohort | 0.925 | 0.925 | 0.925 | 0.970（0.96，0.98） |
|  |  | Testing cohort | 0.875 | 0.888 | 0.887 | 0.919（0.89，0.94） |
| Incus | **RF** | Training cohort | 0.997 | 0.925 | 0.942 | 0.994（0.99，1.00） |
|  |  | Testing cohort | 0.855 | 0.852 | **0.853** | 0.930（0.91，0.95） |
|  | LR | Training cohort | 0.942 | 0.856 | 0.876 | 0.956（0.95，0.97） |
|  |  | Testing cohort | 0.847 | 0.835 | 0.838 | 0.909（0.89，0.92） |
|  | DT | Training cohort | 0.919 | 0.894 | 0.900 | 0.971（0.96，0.98） |
|  |  | Testing cohort | 0.802 | 0.852 | 0.84 | 0.902（0.89，0.92） |
| Stapes | **RF** | Training cohort | 0.975 | 0.894 | 0.915 | 0.989（0.98，0.99） |
|  |  | Testing cohort | 0.852 | 0.867 | **0.863** | 0.931（0.91，0.96） |
|  | LR | Training cohort | 0.923 | 0.864 | 0.879 | 0.960（0.95，0.97） |
|  |  | Testing cohort | 0.852 | 0.865 | 0.862 | 0.922（0.90，0.94） |
|  | DT | Training cohort | 0.850 | 0.883 | 0.875 | 0.946（0.94，0.96） |
|  |  | Testing cohort | 0.845 | 0.853 | 0.851 | 0.902（0.89，0.92） |

Note: AUC, area under the curve; RF, random forest; LR, logistic regression; DT, decision tree; 95%CI, Confidence interval.
